# Validating the measurement of upper limb sensorimotor behavior utilizing a tablet in neurologically intact controls and individuals with chronic stroke

**DOI:** 10.1186/s12984-023-01240-6

**Published:** 2023-09-01

**Authors:** Devin Sean Austin, Makenna J. Dixon, Duncan Thibodeau Tulimieri, Joshua G. A. Cashaback, Jennifer A. Semrau

**Affiliations:** 1 Graduate Program in Biomechanics and Movement Science (BIOMS), University of Delaware, 540 South College Ave. , Newark, DE 19713 USA; 2 Department of Kinesiology and Applied Physiology, University of Delaware, 540 South College Ave. , Newark, DE 19713 USA; 3 Department of Biomedical Engineering, University of Delaware, 540 South College Ave. , Newark, DE 19713 USA

**Keywords:** Stroke, Robotics, Tablet, Sensorimotor, Assessment, Reaching

## Abstract

**Background:**

Intact sensorimotor function of the upper extremity is essential for successfully performing activities of daily living. After a stroke, upper limb function is often compromised and requires rehabilitation. To develop appropriate rehabilitation interventions, sensitive and objective assessments are required. Current clinical measures often lack precision and technological devices (e.g. robotics) that are objective and sensitive to small changes in sensorimotor function are often unsuitable and impractical for performing home-based assessments. Here we developed a portable, tablet-based application capable of quantifying upper limb sensorimotor function after stroke. Our goal was to validate the developed application and accompanying data analysis against previously validated robotic measures of upper limb function in stroke.

**Methods:**

Twenty individuals with stroke, twenty age-matched older controls, and twenty younger controls completed an eight-target Visually Guided Reaching (VGR) task using a Kinarm Robotic Exoskeleton and a Samsung Galaxy Tablet. Participants completed eighty trials of the VGR task on each device, where each trial consisted of making a reaching movement to one of eight pseudorandomly appearing targets. We calculated several outcome parameters capturing various aspects of sensorimotor behavior (e.g., Reaction Time, Initial Direction Error, Max Speed, and Movement Time) from each reaching movement, and our analyses compared metric consistency between devices. We used the previously validated Kinarm Standard Analysis (KSA) and a custom in-house analysis to calculate each outcome parameter.

**Results:**

We observed strong correlations between the KSA and our custom analysis for all outcome parameters within each participant group, indicating our custom analysis accurately replicates the KSA. Minimal differences were observed for between-device comparisons (tablet vs. robot) in our outcome parameters. Additionally, we observed similar correlations for each device when comparing the Fugl-Meyer Assessment (FMA) scores of individuals with stroke to tablet-derived metrics, demonstrating that the tablet can capture clinically-based elements of upper limb impairment.

**Conclusions:**

Tablet devices can accurately assess upper limb sensorimotor function in neurologically intact individuals and individuals with stroke. Our findings validate the use of tablets as a cost-effective and efficient assessment tool for upper-limb function after stroke.

## Introduction

Each year, approximately 800,000 people in the United States experience a stroke, and it is thought that 30-66% of these individuals have persistent upper limb deficits that impact their ability to perform activities of daily living [1–8]. These impairments include hemiparesis, abnormal muscle tone, decreased somatosensation, and impaired coordination of their movements [1, 3, 9–11]. In general, clinical assessments are typically used to quantify these upper limb impairments. While many of these are used in research environments, assessments like the Fugl-Meyer Assessment (FMA) are rarely used in clinical evaluations. Additionally, these assessments rely on participative ratings for quantifying a patient’s impairment, often using narrow ordinal or categorical scales [12]. For example, the FMA is commonly used to quantify upper limb function because it examines a wide range of behaviors such as reaching, grasping, and testing various ranges of motion at several joints [13]. Many behaviors examined in this assessment (e.g. extending metacarpals) are scored on a three-point scale whose categories are represented as; 0-Cannot complete behavior, 1-Behavior cannot be fully completed, and 2-Behavior can be maintained against relatively great resistance. These categories primarily attest to an individual’s overall motor function; however, specific kinematics of the behavior cannot be captured. These measures cannot reliably determine the speed at which the movement was completed, the time it took to respond to a visual cue, or precisely identify kinematic impairments attesting to the quality of the behavior [14]. Knowing these outcomes can give clinicians an indication of the integrity of the sensorimotor system and guide treatments that specifically address the impairment. Additionally, despite being widely used, clinical measures like the FMA can have low interrater reliability and possess known ceiling effects [13–15]. Due to these factors, many clinical assessments are likely unable to capture small, meaningful improvements in function; which may negatively impact treatment efficacy.

The use of robotic technology to assess motor and sensory domains of movement in individuals with stroke has become increasingly popular. Previous studies have validated the ability of robotics to quantify several aspects of upper limb behavior and demonstrate high interrater reliability, high sensitivity, and objectivity [16–21]. Coderre et al. used the KINARM robotic exoskeleton (BKIN Technologies, Kingston, ON) to assess the sensorimotor function of individuals with stroke when completing an eight-target Visually Guided Reaching task (VGR) [16]. This study identified several outcomes from reaching behavior relating to postural control, reactions to visual stimuli, and the quality of movement. The authors reliably identified impairments in 52 individuals with stroke, despite the Chedoke-McMaster Stroke Assessment Scale [22]—a clinical measure designed to assess upper limb impairments in stroke—categorizing several individuals as having no impairments. This suggests that robotic devices can identify upper limb impairments with more sensitivity than clinical measures by capturing precise kinematics that may indicate impairments in individuals with stroke. Other groups have utilized an end-effector robot to quantify sensorimotor impairments in several kinematic metrics (e.g. Reaction Time and Max Speed) that relate to movement quality. Often, these metrics are difficult or impossible to collect with standard clinical assessments, demonstrating the increased utility of robotic devices and their ability to accurately identify upper limb deficits when compared to standard clinical assessments [23–25]. Despite several studies demonstrating the effectiveness of robotic-based assessments for quantifying upper limb impairments in individuals with stroke; these devices are often immobile, expensive, and require significant time and training to operate when used in clinical settings [26].

Tablet-based assessments have the potential to maintain the benefits of both clinical assessments (high mobility, inexpensive, minimal time to operate) and robotic devices (objective, precise kinematics, minimal training needed) while overcoming their limitations; however, the suitability and sensitivity of tablet devices for quantifying upper limb impairments are not yet known [27]. In current stroke-based rehabilitation settings, tablets are primarily used for administrative purposes and assessing cognitive function [28–30]. Kizony et al. used the Dexteria iPad app (Binary Labs) to quantify an individual’s hand dexterity following a stroke [27]. The Dexteria app utilized a tapping task that involved anchoring a participant’s thumb to a set position while using the other digits to tap one of four randomly appearing targets. The average success rate and time needed to complete the task were used to quantify performance during two repetitions of the task; however, these measures cannot indicate overall upper limb integrity because the task exclusively focuses on finger extension. Previous studies have established the feasibility of using tablet-based assessments to quantify hand-motor function and resting tremor in individuals with Parkinson’s Disease [31–33]. To validate the ability of these assessments to quantify upper extremity behavior, studies have correlated performance on these tasks with the Movement Disorder Society-Unified Parkinson’s Disease Rating Scale (UPDRS) which suffers from the same limitations as previously mentioned clinical assessments (i.e., low sensitivity) [34–36]. Recent work has demonstrated the feasibility of tablet-based assessments to quantify impairments in hand-dexterity for a variety of clinical populations; however, these devices have not been compared against commonly used robotic devices whose accuracy and objectivity are validated for capturing upper limb impairments [27, 37].

In this study, we developed a tablet-based application to assess the sensorimotor function of the upper limb by replicating an eight-target center-out reaching task that is commonly used as an upper limb assessment tool in robotic devices [16, 23]. We recruited individuals who have had a stroke and neurologically intact adults to complete a VGR task using both a Kinarm Robotic Exoskeleton and a Samsung Galaxy Tablet. We predicted that the kinematic parameters derived from a proprietary KSA and our custom analysis will be significantly correlated when both analyses are applied to the same datasets. This will indicate that our custom analysis is an accurate replication of the KSA and suitable for both robot and tablet devices. Furthermore, we predicted that the kinematic parameters derived from both devices will be significantly correlated and have no significant within-group differences because the custom analysis will be applied to both devices. We believe that this will indicate consistency across devices and attest to the suitability of the tablet for remote testing of upper limb function in individuals with stroke.

## Methods

### Participant information

Twenty younger controls, twenty older controls, and twenty individuals with stroke participated in this cross-sectional study (Table 1). Control participants were recruited from the immediate regions surrounding the University of Delaware while individuals with stroke were recruited through the University of Delaware Stroke Research Registry. All participants completed two sessions of a Visually Guided Reaching task during a single study visit (details below). The following exclusion criteria were applied to all participants: history of a significant upper-body injury (e.g., shoulder replacement), neurological impairment other than stroke (e.g., Multiple Sclerosis, Parkinson’s Disease), disease that may impact limb sensation (e.g., Diabetic Neuropathy) or to maintain an upright seated posture for > 1 h. Control participants were required to have no previous history of stroke or other neurological injuries. Additionally, individuals with stroke were assessed using the Montreal Cognitive Assessment (MOCA), and if they had moderate to severe (score < 18) cognitive impairment, they were excluded [38]. This study was approved by the University of Delaware Institutional Review Board, and all participants provided informed consent.

**Table 1:** Participant Demographics

	Younger Controls (n = 20)	Older Controls (n = 20)	Individuals with Stroke (n = 20)
Age	24.8 ± 3.56	62.12 ± 9.84	67.1 ± 10.35
Sex	Male: 8 Female: 12	Male: 9 Female:11	Male: 14 Female: 6
Dominant Hand	Left: 1 Right: 19	Left: 1 Right: 19	Left: 4 Right: 15 Ambidextrous: 1
Months Post Stroke	--	--	68.46 ± 34.21
FIM - More Affected	--	--	124.05 ± 1.78
FMA - More Affected	--	--	53.5 ± 15.87
Field Cut	--	--	4
Hemisphere of Stroke	--	--	Left: 9, Right: 11
MOCA	--	--	25.15 ± 3.44
BIT	--	--	142.5 ± 4.04
PPB - More Affected	--	--	6.55 ± 4.67
TLT [0,1,2,3] More Affected	--	--	[19,1,0,0]

Abbreviations: FIM, Functional Independence Measure; FMA, Fugl-Meyer Assessment; MOCA, Montreal Cognitive Assessment; BIT, Behavioral Inattention Test; PPB, Purdue Peg Board; TLT, Thumb Localizing Test. Average ± SD

### Experimental setup

Positional data were recorded using a KINARM Robotic Exoskeleton (Kinarm, Kingston, ON, Canada) and a Samsung Galaxy Tablet S6 (Samsung Technologies, Suwon, Gyeonggi, South Korea) with a Samsung S6 digitizing pen. When completing the task in the robot, participants sat comfortably in the exoskeleton with both arms elevated and supported against gravity. Each segment of the arm was adjusted to raise both upper limbs to a horizontal plane slightly below the shoulder (approximately 80° abduction). An augmented reality system operated in conjunction with the exoskeleton to display targets in the same plane as the participant’s hands while occluding their upper limbs. When completing the task on the tablet, participants sat comfortably at a desk holding a digitizing pen with their elbows comfortably at a 90° angle to the table. The tablet was placed on the desk approximately 5 cm from the table edge and perpendicular to the participant; however, participants could adjust the device at their discretion to a comfortable position. In general, participants did not request that the tablet be moved more than 2 cm in any direction. The tablet displayed visual targets and a cursor that could be controlled by the participant dragging it across the screen with the digitizing pen. Assistive devices for grasping the digitizing pen were offered to individuals with stroke who had issues with grip strength and/or limitations in hand dexterity. These devices include a 7 cm long foam tube (2 cm in diameter and 0.5 cm thick) to increase the overall diameter of the pen, an adaptive utensil cuff that gave participants the option to hold the digitizing pen without using their fingers, and a strip of Dycem grip tape that was wrapped around the pen to reduce the chance of the pen slipping out of the participant’s hand. Two individuals with stroke used both foam tube and grip tape and two used all three adaptive devices. Two individuals with stroke came in with wrist orthotic devices. One chose to wear their orthotic for both tablet and robot tasks, while the other removed it for the entire experiment. Data from both tablet and robot were filtered with a low pass, 10 Hz cutoff Butterworth filter.

### Visually guided reaching (VGR) task

**Fig. 1:**
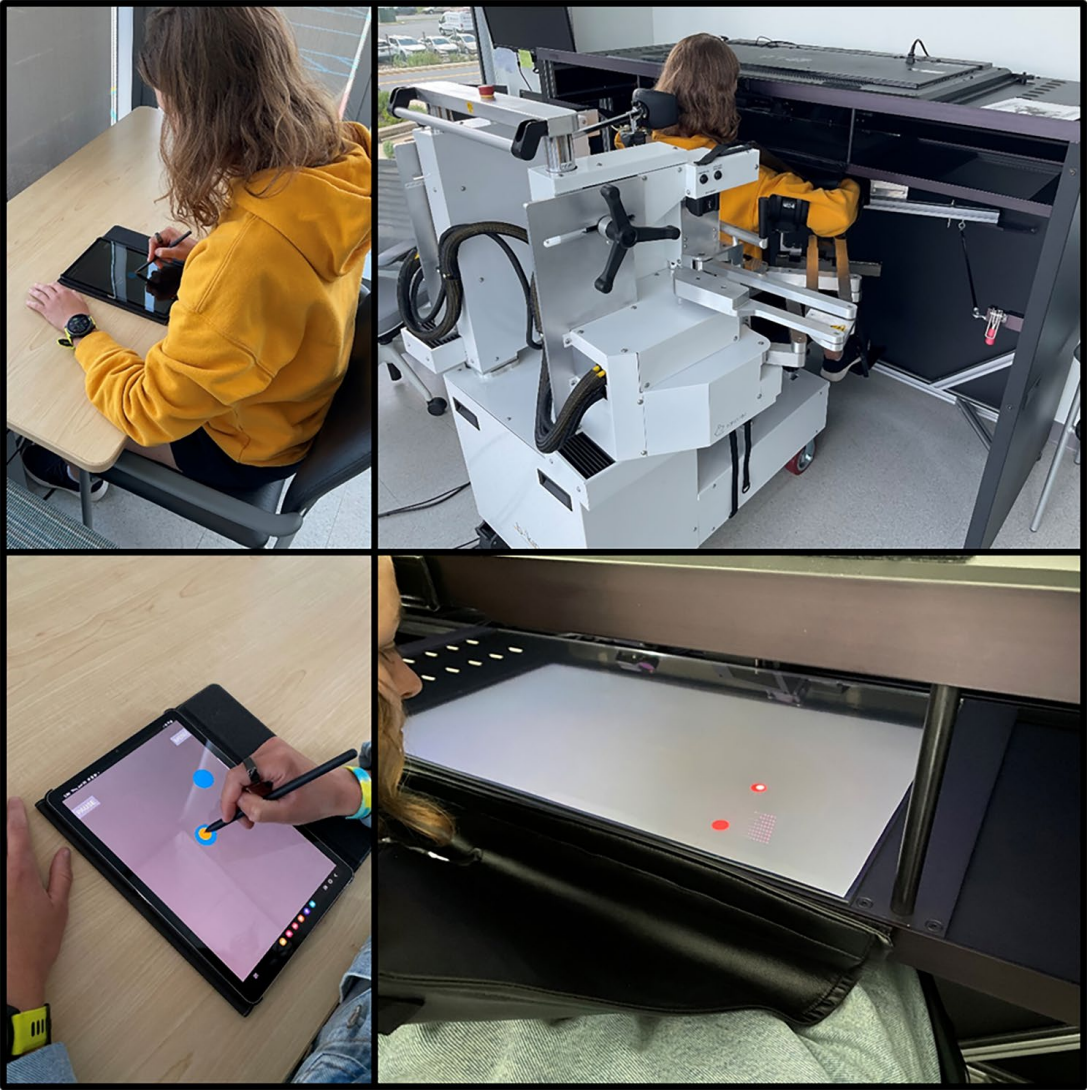
Experimental setups for tablet and robot devices

Participants completed an eight-target VGR task [16] using two different devices: a Kinarm Robotic Exoskeleton and a Samsung Galaxy Tablet (Fig. 1). Participants completed the task twice during a single study visit, once on each device, and device testing order was counterbalanced within each group to control for the effects of practice across devices. The VGR standard task included with the Kinarm Robotic Exoskeleton was used in this study [39]. We created a version of the task suitable for tablet devices with minor alterations due to differences in device capabilities. Notably, the workspace of the robot (200 cm diagonally) was much larger than that of the tablet (26.7 cm diagonally). Therefore, participants made 10 cm reaching movements in the robot and 6 cm movements on the tablet. Additionally, the cursor radius within the robot task was 0.4 cm, which was enlarged on the tablet to 0.6 cm to account for the pen and the participant’s hand occluding the cursor.

Except for the minor differences noted above, outcome measures and instructions for task performance were identical across devices. Participants viewed their fingertip as a cursor and were instructed to hold their finger (digitizing pen with tablet) at a central target for a randomized time (0.75–1.2 s) until one of eight peripheral targets appeared pseudorandomly. Participants were then instructed to move the cursor to the peripheral target as quickly and accurately as possible and hold their position for 1.2 s until the target disappeared; after which they were instructed to return to the central target. The task required individuals to make eight reaches to each target, with catch trials occurring pseudorandomly. During a Catch Trial, participants held the cursor in the central target for a randomized time (0.75–1.2 s) and no peripheral target would appear. The purpose of the catch trials was to ensure participants could not predict the timing between trials. Catch trials never occurred consecutively. From the participant’s perspective, they were unaware a catch trial occurred and stayed within the central target until the next trial appeared. Participants performed a total of 80 trials in the tablet and robot versions of the task, for a total of 160 trials performed within the study visit. Both the robot and tablet assessments took approximately ten minutes; however, the robot portion of the task required an additional ten to fifteen minutes of set up and calibration.

### Clinical assessments

All individuals with stroke underwent a series of clinical assessments by one of two study clinicians, a Physical Therapist and an Occupational Therapist who both have expertise in stroke. Assessments included the Fugl-Meyer Assessment (FMA) to measure arm and hand motor function [13], the Functional Independence Measure (FIM) to measure functional ability [40], the Thumb Localizer Test (TLT) to measure position sense [41], the Behavioral Inattention Test (BIT) to assess for visuospatial neglect [42], the Montreal Cognitive Assessment (MoCA) to assess cognitive function [43], the Purdue Pegboard (PPG) to assess manual dexterity [44], and the Edinburgh handedness Scale to determine handedness [45].

### Kinematic data analysis

We used the previously validated proprietary Kinarm Standard Analysis (KSA) (BKIN Technologies, Kingston, ON) and created a custom analysis to evaluate several measurements of sensorimotor function [16, 21, 39, 46, 47]. To determine each measure on a trial-by-trial basis, upper and lower hand speed thresholds were established to identify the onset and offset of movement, respectively. The upper threshold was based on the 95th percentile of hand speed within each trial during the 0.5 s before the appearance of a peripheral target. Movement onset was identified by finding the time interval between peripheral target illumination and when the cursor leaves the central target. We found the last local minimum in the hand speed that is below the upper-speed threshold. If no local minimum was found, movement onset corresponded to the last observation of hand speed that dropped below the lower speed threshold. The lower speed threshold was based on the median hand speed across all trials during the 0.5 s before the appearance of a peripheral target. Movement offset was identified by examining hand position for the 1.2 s after the participant enters a peripheral target. The algorithm finds the first local minimum in the hand speed that is below the upper-speed threshold. If no local minimum is found, movement offset corresponded to the first instance hand speed dropped below the lower speed threshold. If the participant’s hand speed never dropped below the upper-speed threshold before peripheral target illumination or it took the participant longer than 2s to leave the central target, the trial was noted as a *Start Failure* and was omitted from the analysis. If the hand did not enter the peripheral target then that trial was noted as an *End Failure* and omitted from the analysis. These parameters are akin to parameters evaluated by Coderre and colleagues (No Reaction Time, No Movement End Count) [16].

The offset and onset of the movement were used to determine the following parameters:

1. Reaction Time (s) – the time between peripheral target illumination and the onset of movement. This quantifies an individual’s ability to elicit a movement in response to a visual stimulus.
2. Normalized Movement Time (s/cm) – the total time between movement onset and movement offset divided by the distance between the central and peripheral targets of each device (robot distance: 10 cm, tablet distance: 6 cm).
3. Max Speed (cm/s) – the maximum speed achieved between movement onset and movement offset. This measures the overall movement from a spatial perspective.
4. Initial Direction Error (deg) – the angular deviation between a straight line between movement onset and a peripheral target, and a straight line between movement onset and hand position at the first local minimum post-Max Speed. This characterizes error in the initial phase of the movement.

### Statistical analyses

Our analyses aimed to determine the validity of a tablet device for measuring upper limb kinematics by comparing it to previously validated robotic measurements of upper limb function. To accomplish this, the study had two major goals: (1) to compare the consistency of our calculations of kinematic parameters to the KSA [39] and (2) to compare performance of the VGR task on the tablet and robot using the same kinematic calculations. For analysis comparisons, paired permutation tests were used to determine within-group differences between outcome parameters obtained from the KSA and our custom analyses using the same robot data within participant groups [48–50]. For device comparisons, paired permutation tests were used to determine differences between outcome parameters obtained from our custom analyses for each device within participant groups. For between-group comparisons, non-paired permutation tests were used. To perform the paired permutation tests, we took the difference between the paired samples of each participant, be it for analysis or device comparisons, and took the mean of these differences for our initial test statistic. These differences were then resampled – with replacement – for one million iterations, with each iteration calculating a new test statistic. The new test statistic from each iteration is used to create an approximate distribution of values. We counted several values as or more extreme than the positive and negative instances of our initial test statistic, and divided by the total number of iterations to determine our p-value. For non-paired permutation tests, each set of values used to calculate the initial test statistic is resampled – without replacement – for one million iterations, with each iteration calculating a new test statistic. Spearman correlations were used to determine the relationship between both sets of analyses and devices while measuring their level of agreeability. Additionally, Spearman correlations were used to compare tablet and robot measures to the FMA scores of our individuals with stroke. The following categories were used to classify correlation strength (very weak: rho < 0.19; weak: 0.20 < rho < 0.39; moderate: 0.40 < rho < 0.59; strong: 0.60 < rho < 0.79; very strong: 0.80 < rho < 1.00) [51].

## Results

### Participant characteristics

Our study included three groups of participants, twenty young control, twenty older control, and twenty individuals with stroke. Details on demographic information are in Table 1. As expected, there were significant differences in age between our younger controls (Mean: 24.8 ± 3.56 years) and both the older controls (Mean: 62.12 ± 9.84 years) (p < 0.001) and individuals with stroke (Mean: 67.1 ± 10.35) (p < 0.001); however, there were no age differences between the older controls and individuals with stroke (p = 0.14). Most participants were right-handed (YC = 19, OC = 19, IPS = 15) and one individual with stroke was ambidextrous. All individuals with stroke were at chronic stages (mean Months Post Stroke = 68.46 ± 34.21) and the majority had right hemisphere damage (n = 11). Notably, the average FMA score of this population was 53.4 ± 15.87, indicating a majority of the individuals with stroke had moderate to mild levels of upper limb impairment [52].

### General characteristics of motor behavior

**Fig. 2:**
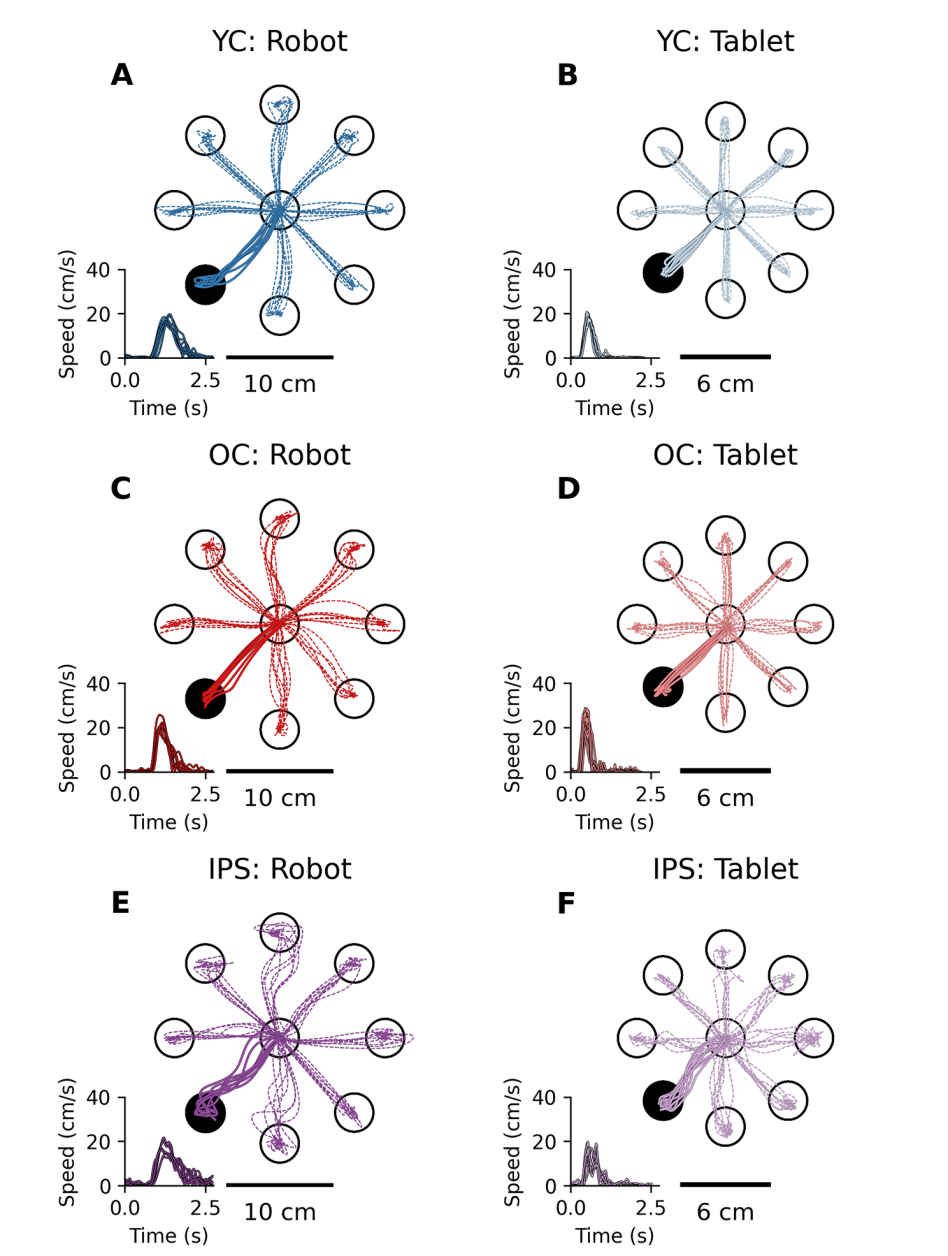
Hand paths for 3 exemplar participants from each group using each device. The lines represent hand paths come from 80 trials of the VGR task with the Kinarm Robotic Exoskeleton (**left column**) and the Samsung Galaxy Tablet (**right column**). **(A)** Performance of a Young Control (YC) participant (24 years old, Right, M) in the Kinarm Robotic Exoskeleton (average Reaction Time = 0.26 s, average Initial Direction Error = 2.03°, Max Speed = 22 cm/s, Movement Time = 1.01 s). **(B)** Performance of the same YC participant in A, but with the VGR tablet task (Reaction Time = 0.28 s, Initial Direction Error = 2.67°, Max Speed = 20.40 cm/s, Movement Time = 0.68 s). **(C)** Performance of an Older Control (OC) participant (69 years old, Right, Female) in the Kinarm Robotic Exoskeleton (Reaction Time = 0.29 s, Initial Direction Error = 2.25°, Max Speed = 19.10 cm/s, Movement Time = 1.10 s). **(D)** Performance of the same OC participant in C, but with the VGR tablet task (Reaction Time = 0.29 s, Initial Direction Error = 2.15°, Max Speed = 25.20 cm/s, Movement Time = 0.48 s). **(E)** Performance of an Individual Post Stroke (IPS) participant (62 Years old, Right Hand Dominant, Male, Right hemisphere of stroke, FMA = 46) in the Kinarm Robotic Exoskeleton (Reaction Time = 0.65 s, Initial Direction Error = 6.62°, Max Speed = 13.36 cm/s, Movement Time = 2.52 s). **(F)** Performance of the same IPS participant in E, but with the VGR tablet task (Reaction Time = 0.30 s, Initial Direction Error = 7.10°, Max Speed = 8.33 cm/s, Movement Time = 1.79 s). Velocity profiles in the lower left corner of each axis represent hand speeds associated with reaches to the black target. The reported results in legend are from the custom VGR analysis

As expected, the hand paths of the exemplar young control for both robot and tablet (Fig. 2A, B**)** were relatively straight with minimal corrective movements needed to reach the target (Robot: Avg. Initial Direction Error = 2.03°, Tablet: Avg. Initial Direction Error = 2.67°). Data obtained from the robot and tablet for the exemplar older control (Fig. 2C, D) exhibited similar reaching characteristics as the young control (OC Robot: Avg. Max Speed = 19 cm/s, Tablet: Avg. Max Speed = 25 cm/s, YC Robot: Avg. Max Speed = 22 cm/s, Tablet: Avg. Max Speed = 20 cm/s). The exemplar individual with stroke moved slower and had similar performance between the robot and tablet (Fig. 2E, F) (Robot: Avg. Max Speed = 13 cm/s, Tablet: Avg. Max Speed = 8 cm/s). On a group level when comparing the performance of younger controls to old controls using non-paired permutation tests, younger controls had shorter Reaction Times (Robot, p = 0.01; Tablet, p = 0.005), faster Max Speeds (Robot, p = 0.029; Tablet, p < 0.001) and shorter Normalized Movement Times (Tablet, p < 0.001). When comparing the performance of older controls to individuals with stroke using non-paired permutation tests, older controls had significantly shorter Reaction Times (Robot, p < 0.001; Tablet, p < 0.001), lower Initial Direction Errors (Robot, p < 0.001; Tablet, p < 0.001) and shorter Normalized Movement Times (Robot, p < 0.001; Tablet, p < 0.001). Additionally, we found that 95% of control participants had one or fewer Start Failures when tested with the robot. We found that seven individuals with stroke were outside this range (11% total trials with Start Failures). Similarly, we found that 95% of control participants had two or fewer Start Failures when tested with the tablet. We found that ten individuals with stroke were outside this range (12% total trials with Start Failures). For End Failures, we found that 95% of control participants had zero End Failures when tested with the robot. We found that seven individuals with stroke were outside this range (3% total trials with End Failures). Similarly, we found that 95% of control participants had two or fewer End Failures when tested with the tablet. We found that six individuals with stroke were outside this range (3% total trials with End Failures).

### Comparison of robotic analyses

**Fig. 3:**
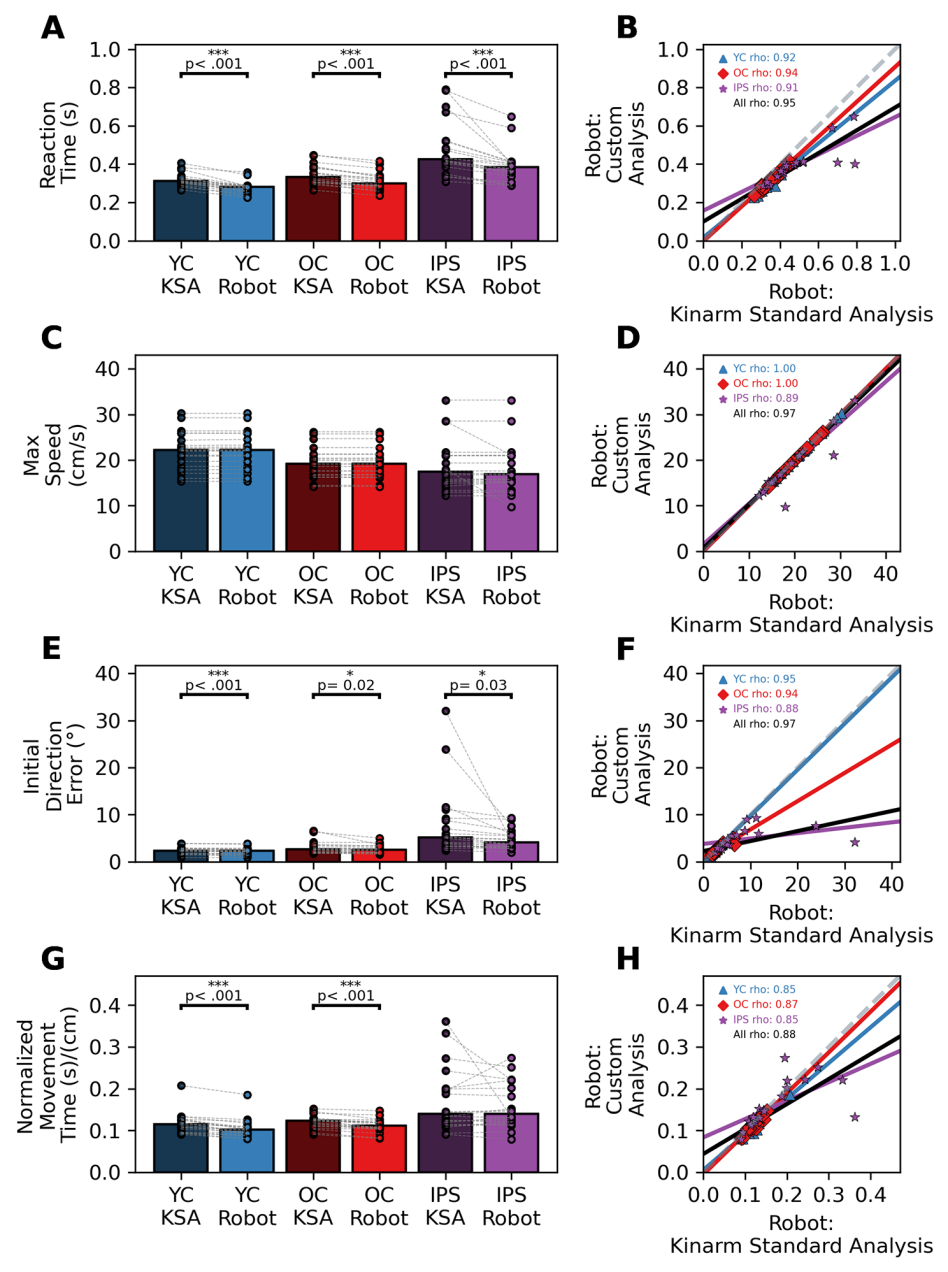
Differences and relationships between outcome measures obtained by the Kinarm Standard Analyses (**KSA**) and our custom analyses (**Robot**) for younger controls (**YC**), older controls (**OC**), and individuals post-stroke (**IPS**). The **left column** shows bar graphs and individual markers to represent both group and individual performance. Dashed lines between markers are used connect participants between devices. Differences between analyses were determined using Paired Permutation Tests. If differences between analyses are observed, p values are shown above the comparison. The **right column** shows correlations between both analyses’ outcome measures for each group. *All* in the figure legend represents collapsed groups. Markers represent an individual’s performance and solid lines represent fits using bootstrapped parameters obtained from performing Ordinary Least Squares on each group. (**A**) Average Reaction Time comparisons between the KSA and custom analysis for YC (KSA: 0.32 s, Robot: 0.28 s), OC (KSA: 0.35 s, Robot: 0.31 s), and IPS (KSA: 0.48 s, Robot: 0.39 s). (**C**) Average Max Speed comparisons between the KSA and custom analysis for YC (KSA: 22.17 cm/s, Robot: 22.18 cm/s), OC (KSA: 19.45 cm/s, Robot: 19.45 cm/s), and IPS (KSA: 18.89 cm/s, Robot: 18.18 cm/s). (**E**) Average Initial Direction Error comparisons between the KSA and custom analysis for YC (KSA: 2.38°, Robot: 2.26°), OC (KSA: 3.02°, Robot: 2.67°), and IPS (KSA: 7.89°, Robot: 4.74°). (**G**) Average Normalized Movement Time comparisons between the KSA and custom analysis for YC (KSA: 0.12 s/cm, Robot: 0.11 s/cm), OC (KSA: 0.12 s/cm, Robot: 0.11 s/cm), and IPS (KSA: 0.17 s/cm, Robot: 0.16 s/cm)

To ensure the correct calculations of our outcome parameters, we used the custom analysis and the Kinarm Standard Analysis (KSA) to compute several kinematic parameters on the same datasets from the Kinarm Robotic Exoskeleton. We observed within-group differences between analyses for **Reaction Time** (YC: p < 0.001, OC: p < 0.001, IPS: p < 0.001), **Initial Direction Error** (YC: p < 0.001, OC: p = 0.02, IPS: p = 0.03), and **Movement Time** (YC: p < 0.001, OC: p < 0.001, IPS: p = 0.36) (Fig. 3A, E, G). While differences were observed within participant groups, each of our outcome parameters was significantly correlated with and across all participants groups (**Reaction Time** YC: rho = 0.92, p < 0.001; OC rho = 0.94, p < 0.001; IPS rho = 0.91, p < 0.001. **Initial Direction Error** YC: rho = 1.0, p < 0.001; OC rho = 1.0, p < 0.001; IPS rho = 0.89, p < 0.001. **Max Speed** YC: rho = 0.95, p < 0.001; OC rho = 0.94, p < 0.001; IPS rho = 0.88, p < 0.001. **Movement Time** YC: rho = 0.85, p < 0.001; OC rho = 0.87, p < 0.001; IPS rho = 0.85, p < 0.001) (Fig. 3B, D, F). This indicates that our custom analysis is similar to the KSA.

### Comparison of robotic and tablet devices

**Fig. 4:**
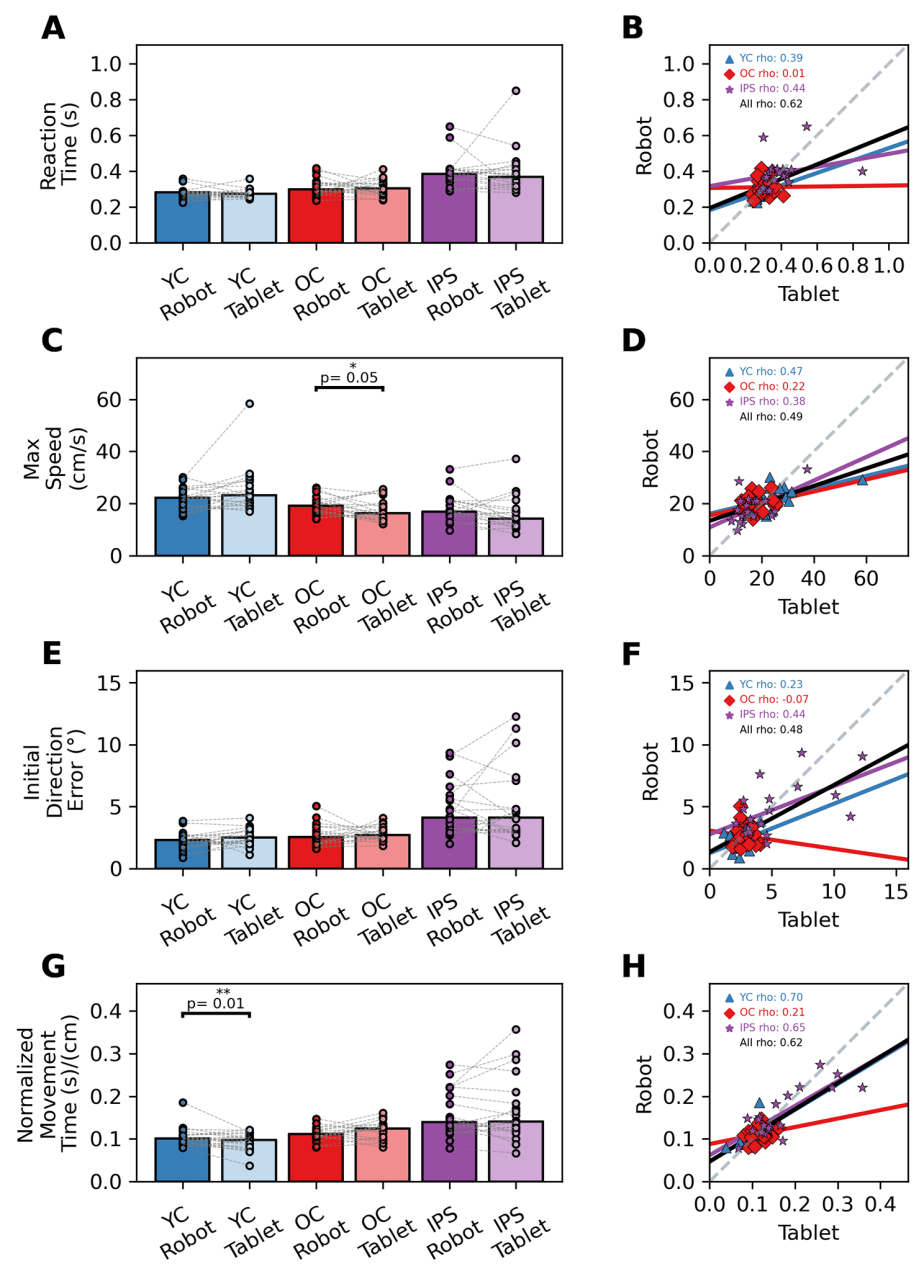
The relationships between outcome measures of the Kinarm Robotic Exoskeleton (**Robot**) and a Samsung Galaxy Tablet (**Tablet)** for younger controls (**YC**), older controls (**OC**), and individuals with stroke (**IPS**). The **left column** shows bar graphs and individual markers to represent both group and individual performance respectfully. Dashed lines between markers are used to track outcome measures calculated across devices. Differences between devices were determined using Paired Permutation Tests. If differences between devices were observed, p values are shown above the comparison. The **right column** shows correlations between both devices’ outcome measures for each group. All in the figure legend represents collapsed groups. Markers represent an individual’s performance and solid lines represent fits using bootstrapped parameters obtained from performing Ordinary Least Squares on each group. (**A**) Average Reaction Time comparisons between devices for YC (Robot: 0.28 s, Tablet: 0.27 s), OC (Robot: 0.31 s, Tablet: 0.31 s), and IPS (Robot: 0.39 s, Tablet: 0.39 s). (**C**) Average Max Speed comparisons between devices for YC (Robot: 22.18 cm/s, Tablet: 25.16 cm/s), OC (Robot: 10.45 cm/s, Tablet: 17.43 cm/s), and IPS (Robot: 18.18 cm/s, Tablet: 16.09). (**E**) Average Initial Direction Error comparisons between devices for YC (Robot: 2.26°, Tablet: 2.53°), OC (Robot: 2.68°, Tablet: 2.82°), and IPS (Robot: 4.74°, Tablet: 5.05°). (**G**) Average Normalized Movement Time comparisons between the KSA and custom analysis for YC (Robot: 0.11 s/cm, Tablet: 0.09 s/cm), OC (Robot: 0.11 s/cm, Tablet: 0.12 s/cm), and IPS (Robot: 0.16 s/cm, Tablet: 0.17 s/cm)

After verifying that our custom analyses accurately quantified several aspects of sensorimotor performance, we applied it to data collected from both the tablet and the robot. Most parameters yielded no significant differences within-group comparisons between robot and tablet devices, demonstrating that the tablet was able to capture upper limb movements similar to that of the robot (**Reaction Time**: YC p > 0.05, OC p > 0.05, IPS p > 0.05; **Max Speed**: YC p > 0.05, OC p = 0.05, IPS p > 0.05; **Initial Direction Error**: YC p > 0.05, OC p > 0.05, IPS p > 0.05; **Movement Time**: YC p = 0.02, OC p > 0.05, IPS p > 0.05). Overall, we observed a wide range of correlations across parameters and between devices. For younger controls, we observed weak to strong correlations across outcome parameters (**Reaction Time** rho = 0.39, p = 0.09; **Max Speed** rho = 0.23, p = 0.34; **Initial Direction Error** rho = 0.47, p = 0.04; **Movement Time** rho = 0.70, p < 0.001). For older controls, we observed relatively weak correlations across outcome parameters (**Reaction Time** r < 0.001, p = 0.97; **Max Speed** rho = -0.07, p = 0.77; **Initial Direction Error** rho = 0.22, p = 0.35; **Movement Time** rho = 0.21, p = 0.37). For the individuals with stroke, we observed weak to strong correlations across outcome parameters (**Reaction Time** rho = 0.39, p = 0.09; **Max Speed** rho = 0.23, p = 0.34; **Initial Direction Error** rho = 0.47, p = 0.04; **Movement Time** rho = 0.70, p < 0.001). Upon collapsing across groups (YC, OC, IPS) we observed moderate to strong correlations between devices for each of our outcome parameters (**Reaction Time**: rho = 0.62, p < 0.001; **Max Speed**: rho = 0.49, p < 0.001; **Initial Direction Error**: rho = 0.49, p < 0.001; **Movement Time**: rho = 0.62, p < 0.001) (Fig. 4, **Right Panel**, ***All***).

### Comparison of devices to FMA scores

**Fig. 5:**
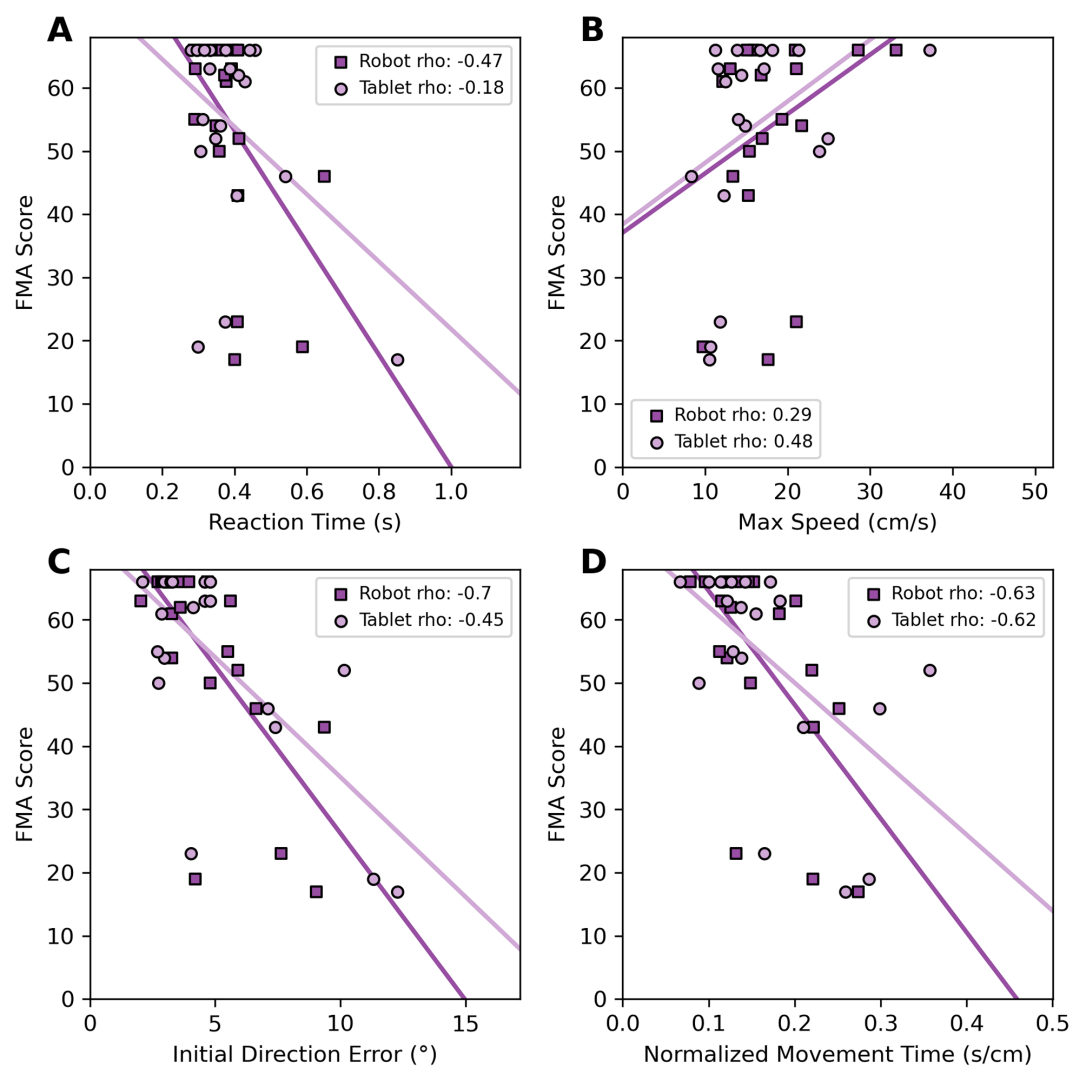
The relationship between outcome parameters obtained from each device and FMA scores for our individuals with stroke. Markers represent individual performance from each device and solid lines represent fits using bootstrapped parameters obtained from performing Ordinary Least Squares on each group. Both the Spearman Correlation Coefficients and associated p values are reported for each device in the legend of the figure. While similarities in device performance are further evidenced by the similar correlations of each FMA score comparison, the influence of the FMA’s ceiling effect is highlighted

To examine the relationship of kinematic data collected from both devices to commonly used clinical measures for the upper limb, we compared data from individuals with stroke to their FMA scores. For Reaction Time, we observed a moderate negative correlation for robot measures (rho = -0.47, p = 0.04) and a weak negative correlation for the tablet measures (rho = -0.18, p = 0.44) (Fig. 5A). For both Initial Direction Error and Movement Time, we observed moderate negative correlations for both robots (**Initial Direction Error**: rho = -0.70, p < 0.001; **Movement Time**: rho = -0.63, p < 0.001) and tablet (**Initial Direction Error**: rho = -0.45, p = 0.05; Movement Time: rho = -0.62, p < 0.001) devices (Fig. 5C, D). These negative correlations indicate that participants with low FMA scores tend to experience increased Reaction Times, Initial Direction Errors, and Movement Times, typically indicative of compromised movement. For Max Speed, we observed a weak positive correlation for the robot measures (rho = 0.29, p = 0.21) and a moderate correlation for the tablet measures (rho = 0.48, p = 0.03) (Fig. 5B), indicating that individuals with lower FMA scores tend to have higher Movement Times.

## Discussion

We determined that a tablet device is an accurate assessment tool for quantifying upper limb sensorimotor function in neurologically intact adults and individuals with stroke. Here, we aimed to (1) test a custom kinematic analysis by comparing it to a VGR analysis that accompanies a suite of KSA [16, 39], and (2) to use this custom analysis to determine whether tablet devices are suitable for testing upper limb sensorimotor function by making direct comparisons with robotic performance using the Kinarm Exoskeleton. We found that (1) our custom analysis was highly comparable to the Kinarm Standard Analysis (KSA), and (2) that there were minimal differences in performance between the Kinarm VGR task and our tablet VGR task. Furthermore, we found that metrics calculated from tablet-based data were significantly related to the FMA scores of our individuals with stroke, further supporting that tablet-based measurements of the upper limb are suitable for quantifying sensorimotor behavior in individuals with stroke.

### Analysis comparisons between Kinarm Standard Analysis and in-house Custom Analysis

The present study demonstrates the effectiveness of our custom analysis for both robotic and tablet devices. Here, we observed considerable overlap between the KSA and our custom analysis when examining the same dataset despite observing significant differences for within-group comparisons in Reaction Time, Initial Direction Error, and Normalized Movement Time (Fig. 3, **left panel**). These differences are likely due to (1) our interpretations of the ambiguous language used in the KSA documentation and/or (2) there is little information describing updates leading to the current version of the KSA [16, 39]. While both reasons stand to maintain the proprietary nature of the KSA, this provides room for error during replication. Notably, the KSA references Coderre et al. for instructions on how to calculate its outcome measures [16]. In the thirteen years since publication, these techniques may have been updated to better capture the kinematic information of individuals with stroke [16]. Despite these discrepancies and possible differences in techniques, our results are within normative ranges for each population and there is a strong correlation between the analyses in each outcome measure regardless of the participant group.

### Device comparisons between Kinarm Robotic Exoskeleton and Samsung Galaxy Tablet

For device comparisons, both robot and tablet data are processed using our validated custom analysis to calculate four measures of sensorimotor function. In addition to the strong correlations of the analyses outlined above, we also observed strong statistical overlap between devices for each within-group comparison. This indicates that a Samsung Galaxy Tablet is a suitable alternative assessment tool for the Kinarm Robotic Exoskeleton. This is evidenced by the minimal within-group differences in our between-device comparisons of each parameter (Fig. 4, **left panel**). There were only two instances where significant differences were observed in our control groups: older controls had a higher Max Speed and younger controls had a higher Movement Time in the robot task. Though minor, these differences may be due to deviations in task execution as well as within-participant variability that may be the result of decreased accuracy (i.e., longer movements) in favor of successful task completion [53]. We were surprised to observe minimal effects of age on reaching as many previous studies have examined this relationship. While some studies have found minimal effects of aging on reaching behavior, most studies have observed decremental effects of aging when examining bimanual movements, reach and grasp behavior requiring coordination and stabilization, as well as movements requiring multi-joint coordination [54–57].

In the Kinarm Robotic Exoskeleton, a participant’s arms are restricted to movements within a 2D plane, limiting the number of strategies that could be utilized to complete the movement. Testing with the tablet was completed with the device resting on a desk with the participant seated directly in front of the tablet. Notably, the tablet itself does not offer gravitational support, and any gravitational support used by participants was provided by the table. Compared to the robot, when participants were tested with the tablet, they could use a variety of strategies to complete the task and were not instructed to hold their limb in a particular posture or engage in a particular reaching behavior beyond the basic task instructions. However, many participants, including individuals with stroke, opted for reaches involving their entire limb during testing on the tablet. The fact that kinematic results did not change as a function of posture in the robot vs. posture when seated with the tablet demonstrates that our method can quantify upper limb function similarly to tablet assessments that exclusively examine hand and finger dexterity [27, 28, 31, 37]. Notably, we observed no significant differences for any outcome measures in our individuals with stroke. This indicates that possible differences in task execution had a non-significant effect on performance and tablets are suitable for upper limb assessments in this patient population.

The idea of using tablets for neurorehabilitation is not necessarily a new one; however, an important distinction should be made. Many studies to date examining the use and implementation of tablets for rehabilitation after stroke have ignored the devices utility in this population. The few studies that have examined the utility of tablets for assessing motor impairment in stroke have mostly focused on manual dexterity or have tested upper limb function within a variable group of neurologic diagnoses that make it difficult to know whether tablets are indeed effective for upper limb assessment in individuals with stroke [27, 37, 58]. We believe our results are the first to demonstrate that tablets can accurately assess upper limb kinematics in individuals with stroke.

### Clinical comparisons

Upon comparing the performance of individuals with stroke on both devices with their FMA scores, we found similar correlations between devices. Additionally, many of our individuals with stroke had a perfect score on the FMA (66). This highlights the well-known ceiling effect that exists with the FMA [13]. Unfortunately, when comparing continuous variables (kinematics) vs. ordinal variables (clinical measures), ceiling effects like those seen with the FMA can impact data analyses, particularly correlations between clinical and kinematic measures. For our Reaction Time measure, we observed a moderate correlation for the robot and no correlation for the tablet. Additionally, one factor that may influence the correlation values of the tablet to FMA scores is that due to limited screen size, the participants’ limbs occasionally obstructed a target in the bottom corner of the screen depending on which hand was used for the task. In these situations, we noted that participants searched for the target, which could potentially lead to an increase in Reaction Time for that trial or they assumed no target appeared.

### Limitations

Several limitations of this study have been previously stated. The first of which is the proprietary nature of the Kinarm Standard Analysis (KSA). Since we were unable to obtain a complete description of the KSA, we assumed the KSA used common calculations found in the literature. Second, each device allowed unique reaching strategies to complete the task. A significant advantage of using the Kinarm Exoskeleton in clinical populations is the ability to assess movement in a gravity-supported environment. This experimental setup is ideal for certain study approaches, but it does not reflect real-world limb movement in the day-to-day life of individuals with stroke. While we did not observe kinematic differences between the tablet and robot for individuals with stroke, we must note that previous work has observed differences in supported vs. unsupported limb movements in individuals with chronic stroke [59]. We must note that a few individuals with stroke who completed the robot portion of the study were fully excluded because they could not complete the tablet portion without full gravitational support (N = 3). This suggests a limitation to the use of tablets as assessment tools, as they may only be suitable for individuals with mild or moderate upper limb impairment. Third, we believe that the ceiling effect of the FMA influences the interpretation of our clinical function (FMA) vs. device (tablet) comparisons. As a result, interpretations focusing on the direct relationship between clinical measures and each device are made with caution due to the potential influence of the ceiling effect that exists for the FMA. Fourth, it is possible that limb kinematics are impacted by the spatial differences (6 cm vs. 10 cm reaches) of the task on each device; longer movements typically require greater movement time, execution, and a greater number of corrective movements; particularly in those with stroke. Future studies should aim to control for the influence of task-based spatial differences between devices by ensuring identical task designs. Lastly, the eight-target visually guided reaching task was not designed with tablet use in mind. As previously stated, some participants had difficulty viewing a target that would appear beneath their hand. Future studies examining upper limb function with a tablet should account for this issue by implementing designs that are user-friendly and consider the influence and limitations of limb posture in the task design.

## Conclusion

We successfully developed a tablet-based assessment that accurately quantifies upper limb sensorimotor function. Both the analysis and task used in the tablet were validated against a common robotic-based assessment where we observed non-significant differences in several outcome measures attesting to various aspects of sensorimotor function. Collectively, our results confirm the potential of tablets as cost-effective and efficient assessment tools for upper-limb function for individuals who have had a stroke. We build on the existing literature of tablet-based assessments for upper limb function in two ways. First, by demonstrating that tablets can capture improved metrics of upper limb function when compared to similar studies examining the use of tablets as assessment devices [27, 37]. Second, to our knowledge, no studies have compared the accuracy of tablets for assessing upper limb kinematics against commonly used precision lab-based robotic devices (e.g., Kinarm Exoskeleton and InMotion). Here, we find that for simple motor assessment of the upper limb, the kinematics captured by the tablet are highly similar to robotics, which suggests that tablets are an excellent candidate for inexpensive and accessible assessments after stroke.

## Data Availability

The datasets used and/or analyzed during the current study are available from the corresponding author on reasonable request.

## Abbreviations

KSA: Kinarm Standard Analysis
VGR: Visually Guided Reaching Task
FMA: Fugl-Meyer Assessment
